# Molecular triaging options for women testing HPV positive with self-collected samples

**DOI:** 10.3389/fonc.2023.1243888

**Published:** 2023-09-22

**Authors:** Katayoun Taghavi, Fanghui Zhao, Laura Downham, Armando Baena, Partha Basu

**Affiliations:** ^1^ Early Detection, Prevention and Infections Branch, International Agency For Research On Cancer (IARC), Lyon, France; ^2^ Department of Cancer Epidemiology, National Cancer Center/Cancer Hospital, Chinese Academy of Medical Sciences, Beijing, China

**Keywords:** Cervical screening, molecular tests, triage, self-collection, cervical cancer elimination

## Abstract

We review developments in molecular triaging options for women who test positive for high-risk human papillomavirus (hrHPV) on self-collected samples in the context of cervical cancer elimination. The World Health Organization (WHO) recommends hrHPV screening as the primary test for cervical screening due to its high sensitivity compared to other screening tests. However, when hrHPV testing is used alone for treatment decisions, a proportion of women of childbearing age receive unnecessary treatments. This provides the incentive to optimize screening regimes to minimize the risk of overtreatment in women of reproductive age. Molecular biomarkers can potentially enhance the accuracy and efficiency of screening and triage. HrHPV testing is currently the only screening test that allows triage with molecular methods using the same sample. Additionally, offering self-collected hrHPV tests to women has been reported to increase screening coverage. This creates an opportunity to focus health resources on linking screen-positive women to diagnosis and treatment. Adding an additional test to the screening algorithm (a triage test) may improve the test’s positive predictive value (PPV) and offer a better balance of benefits and risks for women. Conventional triage methods like cytology and visual inspection with acetic acid (VIA) cannot be performed on self-collected samples and require additional clinic visits and subjective interpretations. Molecular triaging using methods like partial and extended genotyping, methylation tests, detection of E6/E7 proteins, and hrHPV viral load in the same sample as the hrHPV test may improve the prediction of cervical intraepithelial neoplasia grade 2 or worse (CIN2+) and invasive cancer, offering more precise, efficient, and cost-effective screening regimes. More research is needed to determine if self-collected samples are effective and cost-efficient for diverse populations and in comparison to other triage methods. The implementation of molecular triaging could improve screening accuracy and reduce the need for multiple clinical visits. These important factors play a crucial role in achieving the global goal of eliminating cervical cancer as a public health problem.

## 1 Introduction

The World Health Organization (WHO) recommends cervical screening using the high-risk human papillomavirus (hrHPV) test in the general population as well as among women living with HIV (WLWH) (1). With a sensitivity and specificity of 94% (95% CI 89%–97%) and 88% (95% CI 84%–92%), respectively, the test accuracy of hrHPV is better than other screening tests, such as cytology or visual inspection with acetic acid (VIA), for detecting cervical intraepithelial neoplasia grade two or worse (CIN2+) (2, 3). Used alone for screening and treatment decisions, hrHPV testing reduces more cervical cancer-related deaths than these other screening tests (4). Additionally, self-collected and clinician-obtained sampling achieve a similar accuracy when using clinically validated polymerase chain reaction (PCR) for hrHPV DNA detection (3). Offering self-collected hrHPV tests to women has been reported to increase screening coverage (3, 5–8), and allows available health system resources to focus on the effective diagnosis and treatment of the estimated 10% of the general population who may be hrHPV positive (3, 4). Self-sampling strategies were particularly valuable in the COVID-19 context (9).

Ideally, a screening algorithm aims to balance sensitivity and specificity since low specificity may lead to a high proportion of false positives and women being unnecessarily treated (4, 10, 11). Minimizing harm from cervical screening is an important consideration, particularly in young women, due to the potential for adverse reproductive outcomes following precancer treatment (12). As new testing modalities emerge, refined treatment thresholds may be considered. For example, new techniques of histological classification use p16 staining to distinguish CIN2 which is high- and low-grade, reducing unnecessary treatment (13). In settings with sound infrastructure and high adherence to follow-up, treatment thresholds of CIN3 are being investigated among women of childbearing age (14–17). Moreover, the availability of molecular tests enables the detection of a woman’s cancer risk at an earlier phase compared to previous methods. This advancement may allow an extended monitoring period; however, surveillance required for this management is not widely available, especially in low-resource settings.

Screening algorithm fundamentally differ in high and low- and middle-income countries (HIC and LMIC). Generally in HICs, women are only treated when CIN2+ is histologically confirmed and close surveillance is possible for the remaining high-risk women. In LMIC it is more common for treatment to be based on the estimated risk of CIN2+ following one or more screening tests. The tests used in these different screening algorithm fulfil different purposes. Furthermore, in many LMICs, screening and treatment occur on the same day to avoid the transportation barriers and programmatic limitations of arranging the recall of patients. Molecular markers may improve the estimation of cervical cancer risk, which is especially useful for implementing same-day treatment. Molecular biomarkers are also attractive for screening and triage because the automated testing process relies less on training and subjective interpretation (18). Additionally, molecular testing can be performed on clinician-collected and self-collected specimens used for hrHPV testing, removing the need for additional clinic visits. In this review, we describe the advances in molecular triaging options for women testing positive on self-collected samples, highlighting current research gaps and potential future developments in this field.

## 2 HPV testing on self-collected samples in the context of cervical cancer elimination

Cervical cancer is almost completely preventable by vaccination and screening, but worldwide, over 300,000 women die yearly, and 90% live in LMICs (19). These deaths are largely attributable to the inequalities that exist in implementing primary and secondary prevention measures across and within countries (20). The WHO resolution to eliminate cervical cancer aims to reduce global inequalities relating to cervical cancer incidence and mortality (21). This is supported by modelling studies which show that all countries can achieve elimination by the end of the century (22). Elimination is dependent on achieving the following targets; 90% of girls fully vaccinated with the HPV vaccine by the age of 15, 70% of women need screening with a high-performance test (i.e. hrHPV testing) at least twice in their life by the ages of 35 and 45 years, and treating 90% of women who have cervical precancerous lesions or cervical cancer (23). Only 30% of LMICs have implemented national HPV vaccination programs, while they already exist in 80% of HIC (24, 25). Nevertheless, cervical screening will continue to be the most important method of prevention for many decades, because the effects of hrHPV vaccination on cervical cancer incidence and mortality are not immediate (22). Currently, most countries use cytology or VIA as the primary screening test, which are subjective tests (20). The accuracy of these tests can vary significantly depending on the quality of facilities, practitioner training, and quality assurance measures in place (2, 4, 26, 27).

Following the WHO recommendation and the recent European Council recommendations to introduce hrHPV testing for cervical screening, many HICs are transitioning to hrHPV testing and offering self-collection, especially for women who dont participate in screening (28). Given that the overall agreement between DNA hrHPV testing in self-collected versus clinician-collected samples is good (kappa 0.72, 95%CI 0.7–0.8) (8) and samples for self-collected hrHPV testing is well accepted by women in a range of different contexts (3, 8, 29), it is logical to offer this option in screening regimes. A recent modelling study found that self-collection could improve program effectiveness and increase uptake in the general population with only a small compromise in accuracy (7). The COVID-19 pandemic put an unprecedented strain on health services, increasing screening coverage inequalities (9, 30). Self-sampling for hrHPV may offer more efficient and inclusive screening with greater coverage to mitigate the covid-induced delays, reducing the load on the healthcare services (8, 31, 32). The uptake of self-sampling has been especially effective when self-sampling kits are sent directly to women’s homes or offered door-to-door by a health worker (32).

Including a triage test for hrHPV-positive women may improve accuracy of screening and reduce the number of false positives. This is particularly crucial for WLHV due to the high likelihood of hrHPV positivity, and in cases where treatment is based on estimated risk rather than histologically proven CIN2+. In countries where available, cytology triage is currently used after primary hrHPV testing, and studies have shown that this approach can reduce unnecessary treatment and potential harm to women of childbearing age. However, self-collected vaginal swabs are not suitable for evaluating cervical cytology, which limits the benefits of self-collection. WHO recommends triaging hrHPV-positive women with VIA in settings where ensuring quality-assured cytology would be difficult; however, VIA also requires stringent quality assurance to be effective (1, 33). Besides being significantly less sensitive and having all the limitations of a subjective test, VIA triaging also requires an additional clinic visit. Molecular triage may offer a more precise alternative to cytology or VIA triage (7).

## 3 HPV carcinogenesis as the basis for selecting molecular triaging tests

The integration of hrHPV DNA into the host genome is a necessary cause of cervical cancer, leading to the development of malignant cells and immune evasion (34). The process of HPV carcinogenesis begins when hrHPV enters the basal cell layer of the cervical epithelium through microscopic abrasions. Detecting higher levels of hrHPV DNA may suggest a greater risk of carcinogenesis and researchers are now exploring the value quantifying the viral DNA present in cervical samples (35–37). After entering the host cells, the viral DNA integrates into the host DNA, with seven genes expressed in early stages of gene expression, and two genes expressed in later stages. The activation of early genes, E6 and E7, produces vital proteins that result in cellular transformations which may lead to cancer. The E6 gene inhibits p53, enabling the virus to evade apoptosis and accumulate genetic mutations (38, 39). The E7 gene inhibits pRb, causing deregulation of cellular proliferation (38, 39). Although hundreds of HPV genotypes are known, persistent infection with 12 of these are associated with cervical cancer (40–42). Genotypes 16 and 18 have been detected in around 70% of cervical cancer cases, while the risk associated with other hrHPV genotypes is notably lower. This discrepancy in risk is the foundation for using partial and extended genotyping as a molecular triage test. DNA methylation is another molecular option for triage testing. In humans, this test most commonly identifies the addition of a methyl group to the fifth position of the cytosine preceeding guanines (CpG) to form 5-methylcytosine (43–45). DNA methylation of HPV viral genes can also occur following hrHPV infection and cervical neoplasia (43–46). Methylation may change the expression or function of genes but it does not change the genetic code^.44^ Over 100 genes that serve as methylation biomarkers in humans have been tested. In histological samples of CIN2+ and cervical cancer, consistent hypermethylation has been observed in genes such as CADM1, MAL, MIR-124-2, FAM19A4, POU4F3, EPB41L3, PAX1, and SOX1 (18, 46, 47). DNA hypermethylation in the late genes of the hrHPV virus is believed to play a major role in persistent infections. This is because it prevents E2 binding and results in increased E6 and E7 expression (18, 39). Consequently, these changes allow the virus to evade host defence mechanisms and allow the identification of persistent hrHPV infections with the potential to progress to cancer (18, 43, 48). Quantitative methylation-specific PCR and pyrosequencing are the most common methods used for DNA methylation testing. They require a minimal amount of DNA and are highly reproducible (47).

## 4 Evidence supporting available methods of molecular triage

Most hrHPV infections are transient; only 10% of acute hrHPV infections progress to CIN2+ or cervical cancer (49). In some settings, the PPV of hrHPV detection tests can be as low as 5-10% to detect CIN3+ lesions (50). The low PPV of hrHPV testing is also associated with a higher risk of overtreatment and low PPV of hrHPV testing are also associated with a higher risk of over-treatment (3, 26). This is even more problematic in WLWH because hrHPV test positivity may exceed 50% in this high-risk population (51). While more women require additional testing to identify high-grade lesions, colposcopies are inconvenient for women and costly to the health system (3, 26). Using a molecular test for self-collected samples that test positive for hrHPV can reduce the need for women to be recalled for further testing, improving program efficiency and cost-effectiveness. Different molecular triaging options may include (i) partial genotyping, (ii) methylation tests, (iii) hrHPV mRNA detection, (iv) detection of E6/E7 proteins or (v) hrHPV viral load quantification. Additionally, these options may be used in combination.

### 4.1 Partial and extended hrHPV genotyping

#### 4.1.1 Rationale

Advances in commercially available molecular methods have expanded the ability to detect and characterize hrHPV genotypes beyond the research setting (52–55). This has created new possibilities for clinical applications, including risk stratification based on hrHPV genotype as a triage method. The idea of risks stratification based on hrHPV genotype was first reported in 2003 when IARC investigators suggested that women who tested positive for HPV 16, 18, and 45 merits closer surveillance than women infected with other hrHPV genotypes (56). Though most commercially available tests validated for screening detect HPV 16 and 18, certain tests allow the detection of additional common high-risk types (e.g., HPV 45; HPV 31 etc.) in combination or separately. Healthcare providers can tailor interventions to the individual’s risk profile by identifying specific high-risk genotypes, ensuring appropriate surveillance, follow-up, or treatment. These measures may improve cervical cancer prevention and control efforts.

#### 4.1.2 Evidence

Systematic reviews have consistently shown that different hrHPV genotypes are associated with varying risks of CIN2+, and based on this, partial HPV genotyping has already been integrated into national screening guidelines in several countries, including the United States, Canada, Australia, and several European countries (41, 55–60). This is also supported by clinical studies that demonstrate the effectiveness of partial HPV genotyping as a triage test compared with cytology (55, 61–65). For example, the relative risks of CIN2+ following a screening regime of partial HPV genotyping in HPV-positive women in a Japanese cervical screening program was 19.5% (95% CI 12.4–29.4) in women infected with HPV16/18 compared to 5.6% (95% CI 3.1–10.0) in women infected with all 12 high-risk HPV genotypes (61). Another large-scale study among 9,526 women in rural China found that triage strategies among HPV-positive women using HPV16/18 genotyping improved the PPV for detection of CIN2+ by four times. In recent studies, extended hrHPV genotyping beyond HPV16/18 has also demonstrated the potential to improve risk stratification (55, 63–65). For example, in a large US study of 27,037 women with normal cytology, the Onclarity HPV Assay was used. This study found that extended genotyping stratified risk for CIN2+ among women 25+ years with normal cytology and that HPV16 and HPV 31 had the highest risk for CIN2+ (11.6% and 12.1%, respectively) (64). However, using partial genotyping as a triage test may lead to a drop in the sensitivity to 0.71 (95% CI-0.65–0.76), which may be more pronounced with extended genotyping (66, 67).

#### 4.1.3 Implementation considerations

**Figure 1:**
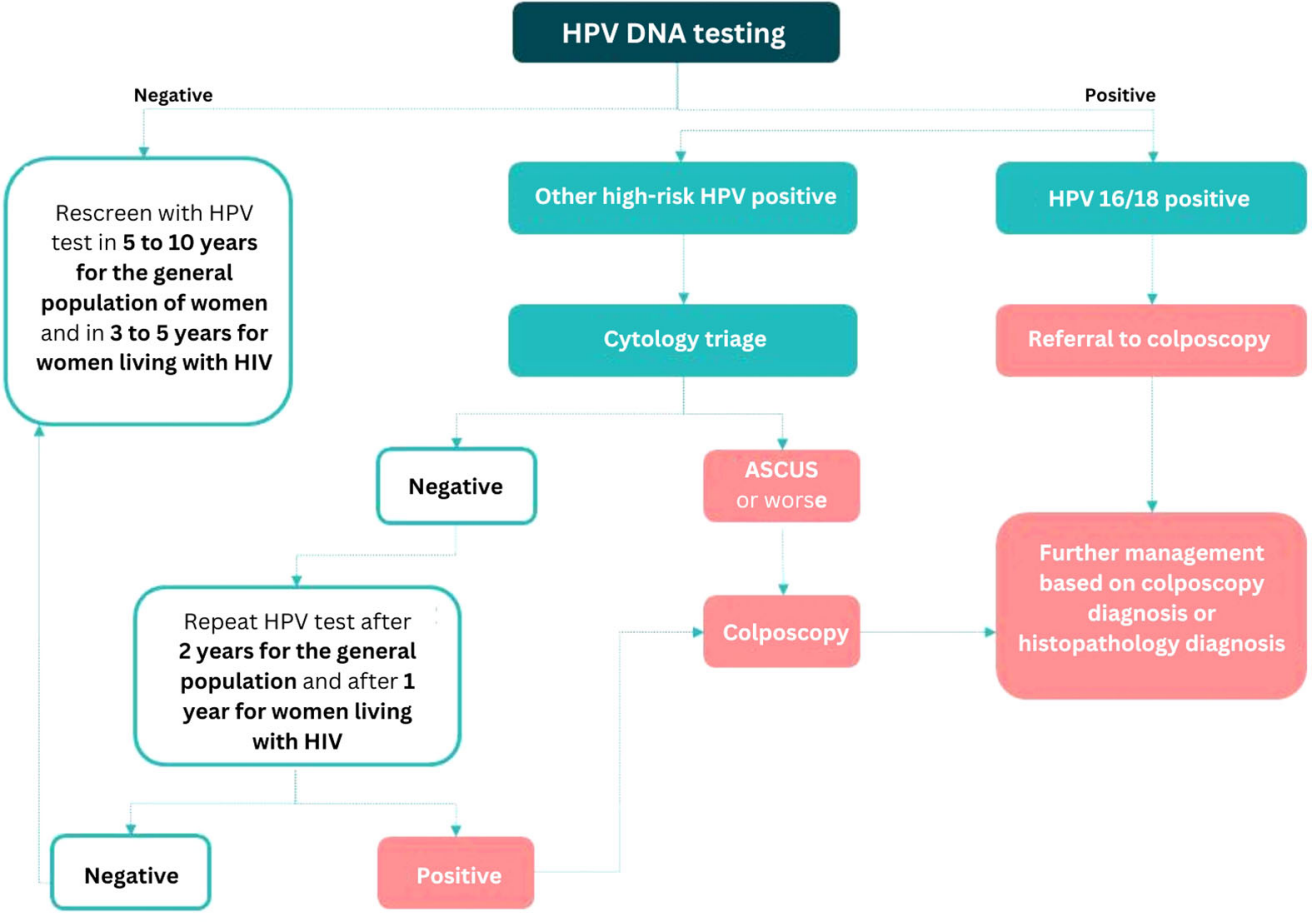
Triaging algorithm for hrHPV-positive women using a combination of partial genotyping and cytology based on the WHO guidelines [Source: Joshi S, Basu P, Lucas E (2023). Using HPV tests for cervical screening and managing hrHPV-positive women – a practical online guide: IARC CancerBase No. 18 [Internet]. Lyon, France: International Agency for Research on Cancer. Available from: https://screening.iarc.fr/atlasHPV.php, accessed on 25 May 2023.

Performing hrHPV genotyping requires access to an appropriate validated test platform that provides genotyping information (at least for HPV 16/18). As discussed earlier, HPV 16/18 as a triage test has to be combined with additional testing (like cytology or VIA for those positive for other hrHPV types), for which the women need to be recalled (**Figure 1**).

### 4.2 DNA methylation of human and viral genes

#### 4.2.1 Rationale

Gene expression and function is affected by DNA methylation. By analyzing the methylation patterns of specific human genes, it is possible to identify early molecular changes associated with the development of CIN2+ lesions (18). Incorporating HPV viral gene methylation provides valuable information on viral activity and persistence. As a triage test, the detection of DNA methylation in both human and viral genes has been found to improve the accuracy of detecting clinically significant precancerous lesions (43).

#### 4.2.2 Evidence

A systematic review of 43 studies demonstrated the association between DNA methylation of several human genes (CADM1, MAL, MIR, EPB41L3, PAX1, SOX1, FAM19A4, and POU4F3) and hrHPV L1/L2 with increasing CIN grade (18, 45). Hypermethylation of these genes is more likely in women with CIN3 compared to CIN1, and they are nearly universally positive in cervical cancer (18). The combination of human and viral gene methylation analysis shows promise for improving triage performance compared to other methods such as hrHPV genotyping and cytology. When used to triage hrHPV+ women, DNA methylation had higher specificity than cytology (relative specificity 1.25, 95% CI 0.99-1.59) and higher sensitivity than HPV16/18 genotyping (relative sensitivity 1.22, 95% CI 1.05-1.42) (18). A study using the DNA-methylation test S5 found the positivity thresholds can be adjusted to alter sensitivity and specificity and tailor the test to the infrastructure capacity in different settings (44). An optimal sensitivity of 96.2% (95% CI 94.3–98.2) was reported (44). Hypermethylation of hrHPV L1/L2 genes has also been assessed separately as biomarkers for CIN2+ and cervical cancer and has better predictive capacity compared with other HPV viral methylation targets with a pooled sensitivity of 77% (95% CI 63%–87%), and specificity 64% (95% CI 55%–71%) (45). However, new targets for HPV methylation have not been thoroughly investigated through whole genome methylation. More broad investigation of methylation patterns may lead to improved triage when compared to hrHPV genotyping, cytology, or their combination (68).

#### 4.2.3 Implementation considerations

While the evidence suggests that DNA methylation of human and viral genes holds promise for improving triage in cervical cancer screening, few tests have been commercialized to date. There is no agreement on which target gene or combination of genes would be ideal. Successful implementation would require further investigation on optimal assays, larger studies on clinical effectiveness, validation studies in diverse populations and cost-effectiveness evaluation.

### 4.3 hrHPV mRNA detection

#### 4.3.1 Rationale

hrHPV mRNA testing specifically detects the presence of viral gene expression, indicating active viral replication and the potential for disease progression (69). When compared to DNA testing, mRNA testing is anticipated to offer more precise detection of persistent infections. This is because mRNA testing targets more advanced stages of the hrHPV pathogenetic pathway. By focusing on viral gene expression, it may be possible to enhance the specificity and PPV of triage tests, which can ultimately reduce the need for unnecessary follow-up procedures for women with temporary hrHPV infections.

#### 4.3.2 Evidence

Detecting hrHPV E6/E7 mRNAs can predict CIN2+ (70, 71). In a large cross-sectional study, it was found that E6/E7 mRNA had high sensitivity (94.4%, 95% CI 89.1–97.3) (72). However, it generates too many positive results for triage and is recommended only for screening in the general population by WHO. There are few studies that examine the accuracy of hrHPV mRNA in self-collected samples or among WLHIV.

#### 4.3.3 Implementation considerations

Due to a paucity of data, especially from longitudinal studies, the WHO has not yet recommended hrHPV mRNA test to be used for self-sampling.

### 4.4 E6/E7 oncoprotein detection

#### 4.4.1 Rationale

The HPV E6 and E7 oncoproteins have a major role in the cervical carcinogenesis, acting as the primary drivers of HPV oncogenic activity (73, 74). As described earlier, they can prevent natural defence against unregulated cell proliferation (such as apoptosis) by deactivating host proteins involved in tumour suppression (such as p53 or pRb) (75–77). Moreover, these oncoproteins have a synergic action which allows them to increase genomic instability and cell mutations, therefore, driving the progression to invasive cancer (78, 79). Detecting these viral oncoproteins could thus mark active and persistent HPV-driven lesions with great potential for carcinogenesis rather than lesions more likely to regress.

#### 4.4.2 Evidence

The OncoE6 Cervical Test detects elevated levels of oncoprotein E6 expressed by HPV16 and HPV18. Across several studies assessing its accuracy, heterogeneous sensitivity estimates were reported for high-grade cervical disease detection, ranging from 54–80%, with specificity estimates ranging from 78-95% when used to triage hrHPV-positive women (accuracy results for CIN2+ were similar to those of CIN3+) (80–84). The OncoE6 Cervical test was also evaluated in a study conducted in Africa to triage hrHPV-positive WLWH and showed a sensitivity of 58.3% (95% CI 30.4–86.2) with a 94.2% (95% CI 91.3–97.2) specificity (85). To improve on this low sensitivity, the OncoE6E7 Cervical test now includes six additional hrHPV types (HPV 45, 31, 33, 35, 52, and 58). One study from China reported promising results using this newly developed triage test showing a higher sensitivity than OncoE6 testing (100% vs 80%) significant difference in specificity (86% vs 92%) for CIN3+ detection, although the study was not adequately powered (83). The benefit of expanding oncoprotein expression to include more HPV genotypes was further established in another multicenter study from Greece and Germany, though this study was limited in sample size (86).

#### 4.4.3 Implementation considerations

Oncoprotein testing has the advantage of being a simple qualitative test based on an immunochromatographic lateral flow format. Results from oncoprotein testing are usually obtained within 3-4 hours, conferring a potential point-of-care use. However, further evaluation studies are required with adequate sample size.

### 4.5 hrHPV viral load estimation

#### 4.5.1 Rationale

The rationale behind viral load estimation lies in the hypothesis that persistent hrHPV infections with higher viral loads are more likely to progress to CIN2+ lesions and, ultimately, to cervical cancer. Viral load testing could offer a semi-quantitative measure of hrHPV infection and may infer increased viral replication.

#### 4.5.2 Evidence

A large study among 39,728 hrHPV+ women found significantly higher detection of CIN2+ with increasing measure of viral load (35). Of great clinical importance, viral load testing identified more than half of CIN2+ lesions missed by colposcopy triage and the level of hrHPV viral load was directly linked to the severity of cervical lesions (35). A threshold of ten relative light units/control (RLU/CO) or higher indicated a suitable criterion for immediate colposcopy, while a viral load between 1 and 10 RLU/CO could be an indication for reflex cytology. This approach optimizes sensitivity and specificity of the test results while managing referral rates effectively (36). A Chinese study involving 2051 women positive for hrHPV on the Cobas4800 test evaluated the role of using cycle threshold (Ct) values for risk stratification. The observed CIN3+ incidence in women with low Ct value (≤ 33.2 for all high-risk types and ≤ 29.6 for high-risk types other than HPV 16) was nine-fold higher than that in HPV-positive women with higher Ct values (87).

#### 4.5.3 Implementation considerations

Currently available PCR assays cannot measure hrHPV viral load, making their implementation in routine clinical settings challenging. Some hrHPV tests like Cobas4800 and Xpert HPV use real-time PCR and indirectly estimate viral loads through Ct values. Ct values indicate the number of cycles needed to detect hrHPV DNA. A low Ct value corresponds to a high viral load, while a high Ct value corresponds to a low viral load. However, only HC2 routinely reports RLU levels, and one must manually extract the Ct values for Cobas or Xpert HPV tests. Further research is required to replicate performance results and comprehend the potential use of different RLU and Ct value cutoffs. In the context of self-sampling, the RLU’s limited performance in detecting CIN2+ implies that it has limited utility as a surrogate measure of hrHPV viral load (3).

## 5 Gaps and future research

Cervical screening is shifting towards objective molecular tests rather than subjective ones like cytology and VIA. Based on the lessons learned from the COVID-19 pandemic, self-collected hrHPV tests are becoming more common, but recalling positive cases for triage tests undermines the purpose of self-collection which is to make screening more accessible and acceptable to women. Further research is needed to better stratify hrHPV-positive women to minimize recalls for colposcopy and treatment to those with the greatest risk of developing cervical cancer.

Self-collected hrHPV testing with molecular triage is promising but requires further research. However, all five options described in this review require robust comparative and longitudinal studies to understand their efficacy in clinically relevant contexts. Studies also need to consider test generalizability and evaluation in different populations. Especially of interest are vaccinated women, women over 65 years who could exit screening programs, WLWH, and women who are receiving post-treatment hrHPV tests (test of cure).

Diverse population data is lacking when reviewing the evidence for partial and extended HPV genotyping. Most of the evidence is derived from specific geographic regions. Where this data is easily available. Further research could be done to understand the potential to stratify the risk in sub-groups of the population, for example, vaccinated women and WLHIV. Evidence on the cost-effectiveness of HPV partial or extended genotyping on self-collected samples and comparisons with other triage methods are essential to inform decision-making and resource allocation.

Longitudinal studies with extended follow-up periods will be useful to assess the predictive value of oncoprotein E6 and E7 in a triage capacity from both self-collected and clinician-obtained samples. None of the evidence cited above includes estimates of test accuracy which are obtained for self-collected HPV samples. So far, the test has been tested in a limited number of geographic settings. There is also a paucity of data to evaluate the clinical utility of oncoprotein E6 and E7 in specific populations, for example, WLHIV. The cost-effectiveness of incorporating E6 and E7 triage using self-collected samples would also need further evaluations in different geographic settings and populations before implementation could be recommended.

The use of DNA methylation as a triage tool is in early stages and still evolving. Currently, the commercially available methylation tests focus on human genes, and experimental tests evaluate viral genes. There remains potential to discover more optimal CpG sites for detecting CIN2+ lesions. Whole genome methylation analysis may provide insight into the breadth of variation in HPV methylation across the genome and enable the identification of new relevant targets. To date, studies evaluating accuracy are very heterogenous and associated with moderate to high risk of bias (45). It is important to consider the differences in study population, their eligibility criteria, the different sampling techniques and tests, as well as the difference in reference verification standards. Testing using self-collected samples and more extensive validation of methylation testing in different geographical settings is required because ethnicity plays an essential role in the epigenome and, consequently, one’s methylation profile. Most studies have been done in Caucasian (43%) or Asian (49%) populations. Whole genome methylation analyses would also enable the identification of common and different cervical cancer-specific markers that may be relevant in different geographical populations, for example, women in sub-Saharan Africa with the highest incidence of cervical cancer worldwide. Detection of hrHPV in first-void urine samples will also be an exciting opportunity to improve screening participation. This is supported by studies finding that it can be as accurate as hrHPV testing on self-collected vaginal samples (88, 89). Some studies are also looking at methylation markers in urine. Testing for hrHPV testing in self-collected vaginal or urine samples and combining a well-validated methylation triage test using the same sample could support the cervical cancer elimination efforts.

Currently, there is no data on the accuracy of testing hrHPV viral load from self-collected samples. However, this may be a consideration in the future. Exploring different thresholds for detection may be relevant for determining the clinical utility of hrHPV viral load as a triage marker. Consistency in sample collection, evaluating and comparing different hrHPV quantification methods, and interpretation criteria is essential to ensure reliable and comparable results. Addressing these gaps in evidence will require further research and studies specifically designed to evaluate each triage test using self-collected samples, comparing it with other triage methods, assessing long-term clinical outcomes, considering cost-effectiveness, and including diverse population sub-sets.

## Author contributions

PB conceptualized the review, contributed to writing and provided critical review and editing. KT, LD, AB and FZ contributed to writing, reviewing and editing. All authors approved the submitted version.
